# The impact of occupational burnout on teaching competence of teachers in Chinese higher vocational colleges: the mediating role of self-efficacy and psychological capital

**DOI:** 10.3389/fpsyg.2026.1791099

**Published:** 2026-05-20

**Authors:** Hailing Xie, Cheng Huang, Qing Zhang

**Affiliations:** 1 School of Education, Yangzhou University, Yangzhou, Jiangsu, China; 2 Department of Basic Courses, Jiangsu College of Tourism, Yangzhou, Jiangsu, China; 3 Hertfordshire College, Changzhou Institute of Technology, Changzhou, Jiangsu, China; 4 School of Law, Yangzhou University, Yangzhou, Jiangsu, China

**Keywords:** higher vocational colleges, occupational burnout, psychological capital, self-efficacy, teaching competence

## Abstract

**Objective:**

To explore the influence mechanism of occupational burnout on the teaching competence of teachers in higher vocational colleges in China, and to verify the separate single mediating and chain mediating effects of self-efficacy and psychological capital between the two variables.

**Methods:**

A total of 1,326 teachers from higher vocational colleges in China were selected as research samples by adopting the stratified random sampling method. Data were collected using the Occupational Burnout Scale, Teacher Self-Efficacy Scale, Psychological Capital Scale, and Teaching Competence Assessment Scale. Data analysis was performed through common method bias test, correlation analysis, regression analysis, as well as structural equation modeling (SEM) combined with the Bootstrap method (with 5,000 resampling iterations).

**Results:**

The structural equation model showed a good fit. Occupational burnout exerted multi-dimensional impacts on the teaching competence of teachers in higher vocational colleges. Specifically, occupational burnout had a significant negative direct effect on teachers’ teaching competence in higher vocational colleges; self-efficacy and psychological capital, respectively, played a significant single mediating role between occupational burnout and teaching competence; and the chain mediating effect of the path “occupational burnout → psychological capital → self-efficacy → teaching competence” was significant.

**Conclusion:**

These findings not only enrich the theoretical research on the relationship between teachers’ occupational burnout and teaching competence, providing empirical support for understanding the association between occupational burnout and teaching competence, but also offer practical guidance for the construction of teacher teams in higher vocational colleges.

## 1 Introduction

In recent years, occupational burnout has become a research hotspot in the field of education, with the problem being particularly serious among teachers in higher vocational colleges. Teachers in higher vocational colleges bear enormous pressure in teaching, research, and student management. Prolonged overwork can lead to a series of burnout symptoms, such as emotional exhaustion, depersonalization, and decreased personal achievement. Occupational burnout not only causes numerous psychological problems for teachers but also impairs their teaching abilities, leading to a decline in educational quality and student academic performance. Therefore, exploring the impact of occupational burnout on the teaching abilities of teachers in higher vocational colleges is of great significance. Research shows that higher self-efficacy and psychological capital help mitigate the negative effects of occupational burnout and improve individual work efficiency (Kim and Burić, 2020). In view of this, this study takes occupational burnout, self-efficacy, and psychological capital as the research starting points to explore the relationships among the three variables and their influence mechanism on the teaching competence of teachers in higher vocational colleges. It is expected to provide theoretical basis and reference strategies for occupational health intervention and teaching quality improvement of teachers in higher vocational colleges.

### 1.1 The relationship between occupational burnout and teachers’ teaching competence

Occupational burnout refers to a series of psychological states such as exhaustion, passivity, and reduced personal accomplishment that arise when individuals engage in a certain occupation for a long time, continuously endure work pressure, and fail to find appropriate ways to relieve such pressure (Maslach and Jackson, 1981). As frontline workers in the education system, teachers bear immense work pressure in their daily work. With the increase of work experience, the intensification of work pressure, and changes in the workplace environment, a growing number of teachers are experiencing varying degrees of occupational burnout. Occupational burnout not only exerts a negative impact on teachers’ own mental health but also has profound effects on their teaching competence, thereby leading to a decline in the overall quality of education and teaching as well as students’ learning efficiency (Oh and Wolf, 2023). An important manifestation of occupational burnout is emotional exhaustion, which refers to a state where teachers feel complete depletion of their physical and emotional energy. Emotional exhaustion causes teachers to lack enthusiasm and passion in their daily teaching activities, which naturally affects the quality of classroom instruction. Severe emotional exhaustion makes it difficult for teachers to reach a high level of teaching competence, and teaching effectiveness also decreases significantly (Skaalvik and Skaalvik, 2010). Depersonalization is another manifestation of burnout, where teachers adopt an indifferent attitude towards students and lack enthusiasm for their daily teaching work. In teachers experiencing depersonalization, students do not receive any form of care or empathy, leading to a cold classroom atmosphere. This negative attitude damages teacher-student relationships and reduces the quality of classroom instruction. Research shows that depersonalization is significantly negatively related with teachers’ job satisfaction and teaching quality (Meyer et al., 2024). Low personal accomplishment is another major aspect of burnout. The loss of personal accomplishment causes teachers to question their teaching abilities and prevents them from gaining recognition and pride from their teaching achievements (Skaalvik and Skaalvik, 2020). This leads to a passive attitude towards teaching, hindering them from engaging in innovative and effective teaching practices (Li et al., 2025). Empirical studies show that low personal accomplishment is often accompanied by lower tolerance and enthusiasm among teachers for solving students’ learning problems, resulting in poor classroom teaching effectiveness.

Digital media platforms have become an important contextual variable influencing the relationship between professional burnout and teaching ability among vocational college teachers. Dixit et al. pointed out that digital media platforms can reduce workload through automated processes, alleviate teachers’ emotional exhaustion, and enhance their teaching self-efficacy. However, if teachers lack the relevant technical application skills, the increased learning costs may exacerbate the erosion of psychological capital by professional burnout (Dixit et al., 2024). The research of Srivastava et al. confirmed that the interactivity of virtual communities in higher education plays a key role. High interactivity can strengthen the optimism and resilience dimensions of teachers’ psychological capital and enhance self-efficacy through peer support, while low interactivity will aggravate teachers’ depersonalization tendency (Srivastava et al., 2024). Baber revealed the potential risks of excessive use of digital media. Its impact on vocational college teachers is a “double-edged sword.” Reasonable use can improve teaching efficiency and enhance teaching achievement, while excessive use can lead to digital burnout, reduce teachers’ psychological capital and self-efficacy, and weaken teaching ability (Baber and Baber, 2026).

In summary, emotional exhaustion, depersonalization, and low personal accomplishment associated with burnout seriously affect teachers’ teaching abilities. Burnout leads to a decline in teachers’ enthusiasm, teaching motivation, and teaching quality, thereby reducing their teaching competence (Song et al., 2023). Therefore, eliminating the negative impact of burnout on teachers is crucial for improving their teaching abilities.

### 1.2 The relationship between occupational burnout and self-efficacy

One of the most famous concepts proposed by psychologist Bandura (1997a,b) is self-efficacy, which refers to an individual’s confidence in their ability to successfully complete a specific task. It represents how people think about and anticipate their ability to effectively complete tasks when facing challenges or goals in a particular situation (Bandura, 1997a). Self-efficacy is particularly important in the field of education. Teachers face a dynamic teaching environment, including various tasks such as knowledge dissemination, classroom management, and student psychological counseling (Dicke et al., 2015). In addition to influencing teachers’ teaching behavior, self-efficacy is also related to their job satisfaction and performance (Zhang et al., 2024). Research shows a significant negative correlation between teachers’ self-efficacy and burnout. Specifically, teachers with high self-efficacy are more likely to effectively cope with obstacles at work and maintain interest and satisfaction in their work (Lauermann and König, 2016). On the other hand, teachers with low self-efficacy are prone to feeling powerless when faced with heavy workloads and begin to experience a series of burnout symptoms, including emotional exhaustion, depersonalization, and reduced personal accomplishment. As teachers become disappointed with the quality of their work and feel emotionally exhausted, a vicious cycle is formed (Skaalvik and Skaalvik, 2011). At the same time, teachers’ self-efficacy not only represents individual characteristics but is also influenced by environmental factors. For example, the school atmosphere and leadership behavior can affect teachers’ self-efficacy (Ambe, 2006). Teachers who receive more support and care from colleagues and supervisors have higher self-efficacy and are therefore able to cope with teaching challenges with greater confidence (Reeves and Hamilton, 2022).

In summary, the occurrence and regulation of teacher burnout are largely influenced by self-efficacy. Higher self-efficacy can enhance teachers’ enthusiasm and confidence in teaching, thus reducing the incidence of burnout. Conversely, lower self-efficacy makes teachers feel powerless and exacerbates burnout (Ju et al., 2015). Therefore, improving teachers’ self-efficacy is considered a key strategy for overcoming burnout problems and improving educational standards. Educational administrators and school principals can help teachers develop a sense of self-efficacy through training, positive feedback, and psychological counseling, thereby reducing the likelihood of burnout (Huang et al., 2019).

### 1.3 The relationship between occupational burnout and psychological capital

Psychological capital (PsyCap) originates from positive psychology and is primarily composed of psychological strengths such as hope, optimism, resilience, and self-efficacy. It helps individuals cope with work stress and problems and enhances psychological resilience (Madrid et al., 2017). Psychological capital is particularly important for teachers. They face challenging teaching tasks, complex work environments, and new educational policies and academic standards, all of which can lead to negative emotions and burnout (Yun et al., 2024). The four dimensions of psychological capital—hope, optimism, resilience, and self-efficacy—play a crucial role in teachers’ professional development and emotional regulation (Ferradás et al., 2019). Hope refers to an individual’s belief that their life goals can be positively achieved. Hopeful teachers usually have clear teaching goals and aspire to achieve them, constantly seeking new ways to solve problems and difficulties at work. Optimism means having a positive attitude towards the future; optimistic teachers usually believe that their courses will ultimately be successful, and this belief keeps them highly motivated and allows them to persevere even when faced with setbacks. Resilience refers to the ability to recover quickly and move forward in the face of adversity, failure, or setbacks (Ma, 2023). Teachers’ resilience enables them to overcome numerous challenges in the teaching process and teach effectively. Finally, self-efficacy refers to a person’s belief in their ability to complete the task at hand. High-performing teachers usually believe that they have excellent teaching abilities and can better cope with various classroom challenges (Hazan-Liran and Karni-Vizer, 2023). In addition to directly influencing teachers’ work motivation and efficiency, psychological capital (PsyCap) can also indirectly reduce teachers’ burnout levels by regulating self-efficacy (Zhao et al., 2022). This means that teachers with high psychological capital are usually more confident in dealing with teaching challenges, which particularly helps to enhance their self-efficacy. Once teachers feel that they can effectively fulfill their teaching responsibilities, their self-efficacy will be higher, thus reducing emotional exhaustion and burnout. Therefore, both burnout and teaching skills are related to the direct and indirect effects of psychological capital: on the one hand, it directly affects teachers’ emotions; on the other hand, it improves teaching quality by enhancing teachers’ self-efficacy (Pu et al., 2016). Therefore, this means that enhancing psychological capital has become an effective way to address teacher burnout. Existing research shows that interventions focusing on psychological capital can enhance teachers’ stress resilience and self-efficacy, thereby mitigating the negative effects of burnout (Zhang Q. et al., 2023). The development of teachers’ psychological capital can also be promoted through methods such as mental health training and providing positive feedback. These measures not only help reduce teachers’ work stress but also enhance their self-confidence and work efficiency.

In summary, psychological capital is one of the key factors influencing teacher burnout and their teaching abilities. Enhancing teachers’ psychological capital helps reduce the level of teacher burnout and improve their teaching performance.

### 1.4 The mediating roles of self-efficacy and psychological capital

The Mediating Role and chain mediating Role of self-efficacy and psychological capital on occupational burnout and teaching competence have been among the most discussed topics in educational research in recent years (Zhang W. et al., 2023).

Self-efficacy plays an mediating role in occupational burnout and teaching competence. Self-efficacy is a contextual cognitive belief targeted at teaching tasks, representing teachers’ independent judgment of their ability to complete specific teaching tasks. As a specific belief variable in the teaching context (Dexter and Wall, 2021). High self-efficacy enables teachers to engage in teaching with greater confidence, thereby better helping them solve problems that arise in the teaching process and effectively reducing the impact of occupational burnout on teaching competence.

Psychological capital plays an mediating role in occupational burnout and teaching competence (Liu and Du, 2024). Psychological capital is a holistic, trait-based positive psychological resource at the individual level. This study defines its core dimensions as hope, optimism, resilience, and self-confidence, which reflect the overall level of positive psychological resources in the form of a first-order four-factor model (Reichard et al., 2024). Occupational burnout directly affects teachers’ psychological capital level, which in turn affects their teaching competence. High-level psychological capital can enhance teachers’ resilience and help them maintain a positive work attitude, enabling them to ensure high-quality teaching even with heavy teaching workloads.

Psychological Capital (covering four core dimensions: hope, optimism, resilience and self-confidence) and Self-Efficacy are key variables in the research on teachers’ occupational psychology. Apart from exerting mediating role, they also play a significant chain mediating role (Bi and Ye, 2021). Chain mediating role is a special form of the multiple mediating role and is fundamentally different from parallel mediating role. In parallel mediating, each mediator functions independently. By contrast, the core characteristic of chain mediating role is that multiple mediators transmit causal role sequentially in a fixed order, forming a continuous causal pathway: “independent variable → Mediator 1 → Mediator 2 → dependent variable” (Yan et al., 2025). In this study on teachers, the chain mediating role path is presented as “Occupational Burnout → Psychological Capital → Self-Efficacy → Teaching Competence”, which reflects an obvious sequential transmission logic: Occupational Burnout first depletes teachers’ Psychological Capital, thereby weakening their teaching Self-Efficacy, and ultimately causes substantial impairment to their Teaching Competence.

Psychological Capital is a fundamental positive psychological resource at the individual level, whereas Self-Efficacy refers to teachers’ specific cognitive beliefs about their own Teaching Competence. These two constructs follow the internal hierarchical logic of “resource reserve → belief formation → behavioral performance” (Jiang and Yuan, 2025). The variable sequence of “Psychological Capital → Self-Efficacy” in this study is not subjectively defined but grounded in solid theoretical support, mainly based on two major theories: Conservation of Resources Theory (COR): Psychological Capital falls into the category of “resource reserve” and acts as the basic positive psychological resource owned by teachers; Self-Efficacy, developed on the basis of resource reserves and cognitive judgment of personal abilities, belongs to the level of “belief formation”; Teaching Competence is the practical behavioral performance transformed from such beliefs. In accordance with the core logic of Conservation of Resources Theory, resources precede beliefs, and beliefs precede behaviors. Hence, the sequence of “Psychological Capital → Self-Efficacy” fully conforms to the hierarchical transmission logic of “resource reserve → belief formation → behavioral performance “, which is an inevitable conclusion in theory (Hobfoll, 1989). Social Cognitive Theory: The formation of Self-Efficacy relies heavily on the support of individual psychological resources. The dimensions of Psychological Capital including hope, optimism and resilience constitute the precondition psychological foundation for the formation of teachers’ teaching Self-Efficacy. There exists a stable causal relationship between the preceding independent variable and the subsequent dependent variable, and a high level of Psychological Capital can directly strengthen teachers’ positive beliefs in their own Teaching Competence (Bandura, 1977b).

Compared with the mediating role, the chain mediating role has irreplaceable core value in interpreting the relationship between teachers’ Occupational Burnout and Teaching Competence. The mediating role can only separately explain the independent mediating role of Psychological Capital or Self-Efficacy, and it cannot reveal the hierarchical transmission relationship between the two or present the complete action process of variables. On the contrary, the chain mediating role can fully demonstrate the continuous psychological transmission chain of “Occupational Burnout depletes psychological resources → weakens beliefs in teaching competence → reduces teaching behavioral performance”. It is more consistent with the real psychological mechanism of how Occupational Burnout influences teachers’ Teaching Competence, can comprehensively and profoundly explain the complex relationships among variables, and effectively makes up for the explanatory deficiencies of the mediating role.

In summary, Self-Efficacy and Psychological Capital can serve as individual mediators to play a Mediating Role in alleviating the adverse effects of Occupational Burnout on Teaching Competence, and they can also exert a Chain Mediating Role in the chain structure of Occupational Burnout → Psychological Capital → Self-Efficacy → Teaching Competence. High levels of Self-Efficacy enhance teachers’ confidence and problem-solving abilities, while high levels of Psychological Capital strengthen their resilience and stress management skills. The combination of these two factors can reduce the negative impact of Occupational Burnout on teachers.

## 2 Model and hypotheses

### 2.1 Hypotheses

*H1:* Occupational Burnout has a negative impact on the teaching competence of teachers in higher vocational colleges.

*H2:* Psychological capital plays a mediating role between Occupational Burnout and teaching competence.

*H3:* Self-efficacy plays a mediating role between Occupational Burnout and teaching competence.

*H4:* Self-efficacy and psychological capital exert a chain mediating effect between Occupational Burnout and teaching competence.

### 2.2 Model

**Figure 1:**
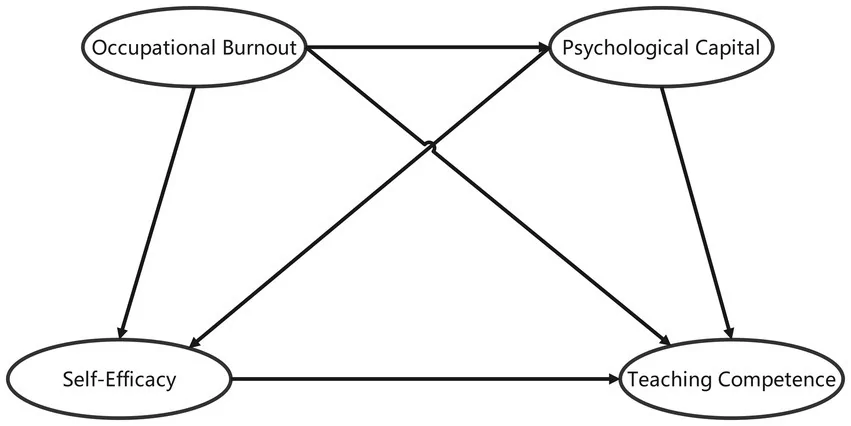
Theoretical model diagram.

As can be seen from Figure 1, In this study, the four core variables—occupational burnout, psychological capital, self-efficacy, and teaching competence—are all modeled as first-order multi-dimensional structures within the framework of Structural Equation Modeling (SEM).

Occupational burnout: A first-order three-factor structure (emotional exhaustion, depersonalization, reduced personal accomplishment), classified according to the classic dimensions of the Maslach Burnout Inventory.

Psychological capital: A first-order four-factor structure (hope, optimism, resilience, self-efficacy), established based on the core dimensions of the Psychological Capital Questionnaire (PCQ).

Self-efficacy: A first-order three-factor structure (classroom management, instructional strategies, student academic achievement), divided according to the dimensions of the Teachers’ Sense of Efficacy Scale (TSES).

Teaching competence: A first-order four-factor structure (classroom instruction, student engagement, educational innovation, teaching quality), defined based on the dimensions of the Teaching Competence Assessment Scale (TCAS).

This modeling approach follows the general norms of quantitative research in education and psychology. It can accurately reflect the independent contribution of each dimension to the overall construct, and is highly consistent with the dimensional characteristics of the variables in this study and the path analysis logic of the chained mediation. The model fitting results have also verified the rationality of this specification.

## 3 Methods

### 3.1 Participants

The research focuses on the teaching abilities of in-service teachers in Chinese higher vocational colleges. A stratified random sampling method was used to select teachers from higher vocational colleges in different provinces across China. This sampling strategy ensured that the sample represented teachers from diverse regional backgrounds, educational backgrounds, and types of institutions. Furthermore, the researchers selected teachers from various academic disciplines, including science and engineering, fine arts, humanities and law, social sciences, and vocational skills, to broaden the applicability of the research findings. This study strictly adheres to the ethical standards stipulated in the Declaration of Helsinki (1964) and its subsequent revisions.

### 3.2 Measurement tools

This study employed a series of standardized assessment tools to conduct an in-depth investigation into teacher burnout and its determinants (such as teacher self-efficacy, teacher psychological capital, and teacher teaching ability). Each scale demonstrated extremely high reliability, validity, and goodness of fit. In this study, the three dimensions of occupational burnout (emotional exhaustion, depersonalization, reduced personal accomplishment), four dimensions of psychological capital (hope, optimism, resilience, self-efficacy), three dimensions of self-efficacy (classroom management, instructional strategies, student academic achievement), and four dimensions of teaching competence (classroom teaching, student engagement, educational innovation, teaching quality) are all defined as first-order multidimensional constructs. The dimensional division of each variable is derived from the classic theoretical frameworks of mature scales; meanwhile, this modeling method conforms to the general norms of quantitative research in the fields of education and psychology and is consistent with similar empirical studies.

#### 3.2.1 Maslach Burnout Inventory

The Maslach Burnout Inventory (MBI) is a traditional test developed by Maslach and Jackson (1981) to assess occupational burnout. The questionnaire includes three dimensions: emotional exhaustion, depersonalization, and reduced personal accomplishment. This study focuses on these three dimensions—emotional exhaustion, depersonalization, and reduced personal accomplishment—to measure the occupational burnout experienced by teachers in their work. The scale uses a 5-point Likert scale, where 1 indicates strong disagreement and 5 indicates strong agreement. Reliability and Validity: The overall scale coefficients (*α* coefficients) for the three dimensions of emotional exhaustion, depersonalization, and reduced personal accomplishment were 0.91, 0.93, and 0.85, respectively. The model fit indices from confirmatory factor analysis (CFA) were: χ^2^/df = 2.59, CFI = 0.92, TLI = 0.91, RMSEA = 0.045, indicating a good model fit.

#### 3.2.2 The Teacher Self-Efficacy Scale

Teacher Self-Efficacy Scale (TSES) is a measurement instrument created by Tschannen-Moran and Hoy (2001) to measure teacher self-efficacy in the context of classroom teaching. It consists of three sub-dimensions: classroom management, instructional strategy, and student academic performance. High self-efficacy teachers will be effective in dealing with different problems and challenges they face in the classroom and hence improve their teaching skills and performance. Reliability and Validity: Cronbach’s alpha of the total scale was 0.93, the alpha of classroom management had a value of 0.91, while the alpha of the instructional strategies and the students’ academic achievement were 0.89. As per the results of Confirmatory Factor Analysis (CFA) the values of the chi-square divided by the degrees of freedom were 2.77, CFI was 0.93, TLI was 0.92, and RMSEA is 0.048 which means that the model had a good fit.

#### 3.2.3 The Psychological Capital Questionnaire

Psychological Capital Questionnaire (PCQ) is a measurement instrument created by Luthans et al. (2010) to assess the level of psychological capital of individuals. These are Hope, Optimism, Resilience, and Self-efficacy (four subscales). With psychological capital an individual can have an adequate mental state as well as appropriate coping strategies especially during stress and challenges. It also utilizes a five -point Likert scale in scoring which indicates that 1 means strongly disagree and 5 means strongly agree. Reliability and Validity: The Cronbach’s alpha of the total scale was 0.94 and 0.90 on Hope, 0.86 in Optimism, 0.88 in Resilience and 0.91 in Self-efficacy. The findings of Confirmatory Factor Analysis (CFA) indicated that there were acceptable levels of construct validity; that is, *χ* 2/d *f* = 2.42, CFI = 0.96, TLI = 0.95, and RMSEA = 0.039.

#### 3.2.4 Teaching Competence Assessment Scale

Berk (2005) formulated a broad measure for assessing the teaching competence of teachers. This scale consisted of the synthesis of 12 technical ways to assess the quality of teaching of the teacher, such as the assessment of students, the assessment of peers, self-assessment, classroom video recording, student interview, etc. These technical methods have both quantitative and qualitative assessment techniques and it has been suggested that mixed data collection strategies are able to enhance the representative nature and accuracy of teaching quality evaluations. Reliability and Validity: Overall scale: The Cronbach’s alpha value of the total scale was 0.92 and the values of classroom teaching, student engagement, educational innovation, and teaching quality were 0.89, 0.87, 0.88, respectively. The findings of Confirmatory Factor Analysis (CFA) indicated that χ2/df = 2.60, CFI = 0.94, TLI = 0.93 and RMSEA = 0.046 which implies excellent model fit and construct validity.

The all the above-mentioned scales reported to be very reliable and valid as well as having great construct validity, which guaranteed its applicability to teachers in higher vocational college and formed a trustworthy basis of information that would then be used in further data processing and research.

### 3.3 Data collection and analysis methods

#### 3.3.1 Data collection

The present study has followed rigorous academic ethics and also the idea of humanistic care. Before conducting the study, the Institutional Ethics Review Panel approved it. All the participating teachers were given an Informed Consent Form before they participated in the study that had all significant details such as objective of the study, data usage limits, confidentiality safeguards, right to freely take part, and withdrawing procedures. Only once teachers read and signed the form, they were permitted to complete the questionnaire. To achieve representativeness of the sample, 1,326 teachers who were employed in various higher vocational colleges of the country in various regions and disciplines were chosen as the research subjects. This survey took both online and offline approaches to facilitate participation of all forms of teachers. The number of questionnaires collected was about 1,425. The final number of valid questionnaires was found to be 1,326 (after discarding the invalid ones), giving an effective recovery rate of 93.23%. Missing values and logical inconsistent data were sorted out to exclude them, which guaranteed the validity and credibility of data. It can be concluded that the data collected in this research are comprehensive, representative and accurate and will provide the solid data support of many future statistic analyses. In the actual survey process, no negative items were reverse-coded, all statistical analyses were conducted based on the raw scores, negative associations were the true relationships between variables, and coding bias interference was excluded.

#### 3.3.2 Data analysis methods

Descriptive Statistical Analysis: A descriptive statistical analysis based on basic demographic variables concerning teachers (gender, age, educational history, and years spent teaching) would provide an overall picture of the study sample. To have an approximate understanding of the overall status of teachers regarding Occupational Burnout, self-efficacy, psychological capital, and teaching competence, some pertinent indicators like mean and variance were computed with regard to the scores on each scale.

Correlation Analysis: In order to investigate how the variable, Occupational Burnout is associated with other variables such as self-efficacy, psychological capital and teaching competence, the Pearson correlation analysis was used as an instrument to measure the correlations among the variables. The correlation analysis helped to understand the fundamental linear relationships between the variables.

The mediation effect analysis: Structural Equation Modeling (SEM), was used to investigate the mediating role of both self-efficacy, and psychological capital on the connection between Occupation Burnout and teaching competence of teachers. Path analysis was also used as a form of mediation effect analysis to determine how Occupational Burnout influences teaching competence with the help of self-efficacy and psychological capital as mediators. Moreover, the Bootstrap technique was applied in the mediation effect analysis to confirm the mediating effects and thus improve the reliability of the findings.

SPSS and Amos software were used to analyze the data. In particular, SPSS was primarily applied to descriptive statistics, correlation analysis, and reliability and validity tests; Amos was primarily used to conduct a path analysis of the SEM and confirm mediating effects.

## 4 Results

### 4.1 Common method bias test

In this study, self-reported questionnaires were used to measure occupational burnout, psychological capital (PsyCap), self-efficacy, and teaching competence. To prevent potential common method bias (CMB) from affecting the research results, a combination of procedural control and technical testing was adopted for CMB prevention and detection. During the survey, measures such as anonymity, random arrangement of scale items, and emphasis on data being used solely for research purposes were implemented to reduce participants’ social desirability responses. At the technical testing level, Harman’s single-factor test was used to conduct unrotated exploratory factor analysis (EFA) on all variable items. The results showed that a total of four common factors with eigenvalues greater than 1 were extracted. The variance contribution rate of the first common factor was 32.67%, which was lower than the critical value of 40%. Meanwhile, the cumulative variance explanation rate of the four common factors reached 68.92%. Further tests revealed no phenomenon where a single factor explained the majority of the variance. Thus, the CMB issue in the data of this study is within an acceptable range and is insufficient to affect the test results of the relationships between variables, indicating that the research conclusions have good reliability.

### 4.2 Descriptive statistics and correlation analysis

To verify the relationships between occupational burnout, self-efficacy, psychological capital (PsyCap), and the teaching competence of teachers in higher vocational colleges, this study conducted Pearson correlation analysis on a sample of 1,326 teachers. Presented below are the means, standard deviations, and correlation coefficients of the main variables, which provide basic data support for the subsequent mediation tests.

As shown in Table 1, significant correlations were observed among teachers’ occupational burnout, self-efficacy, psychological capital (PsyCap), and teaching competence. Descriptive statistical analysis revealed that the score of teachers’ occupational burnout was (2.48 ± 0.59), falling in the low-to-moderate level; while self-efficacy (3.22 ± 0.53), PsyCap (3.31 ± 0.55), and teaching competence (3.15 ± 0.61) were all at the moderate-to-high level. Correlation analysis indicated that occupational burnout was significantly negatively related with self-efficacy, PsyCap, and teaching competence (*r* = −0.40, −0.44, −0.62 respectively, all *p* < 0.01). Self-efficacy was significantly positively related with PsyCap and teaching competence (*r* = 0.49, r = 0.46 *p* < 0.01), and PsyCap was significantly positively related with teaching competence (*r* = 0.52, *p* < 0.01). The above results partially confirm the research hypotheses, laying a foundation for the subsequent mediation effect test to a certain extent. They suggest that occupational burnout leads to a decline in teachers’ teaching competence by reducing self-efficacy and weakening PsyCap, and also provide certain data support for the implementation of subsequent intervention measures.

**Table 1:** Correlation analysis of variables and their dimensions.

Variables	Mean ± SD	1. Occupational burnout	2. Self-efficacy	3. Psychological capital (PsyCap)	4. Teaching competence
1. Occupational burnout	2.48 ± 0.59	1			
2. Self-efficacy	3.22 ± 0.53	−0.40**	1		
3. Psychological capital (PsyCap)	3.31 ± 0.55	−0.44**	0.49**	1	
4. Teaching competence	3.15 ± 0.61	−0.62**	0.46**	0.52**	1

** Indicates *p* < 0.01, and the results are statistically significant.

### 4.3 Regression coefficient analysis

To test the mediating paths among variables and the influence mechanism on the teaching competence of teachers in higher vocational colleges, this study took 1,326 teachers as research subjects and used multiple regression to examine the predictive relationships between variables. The results are shown in Table 2.

**Table 2:** Results of multiple regression analysis of variables.

Dependent variables	Predictor variables	Overall model fit index (*R*^2^)	Standardized regression coefficient (*β*)	Significance of regression coefficient (t-value)
Psychological capital (PsyCap)	Occupational burnout	0.19	−0.44	−15.12***
Self-efficacy	Occupational burnout	0.28	−0.24	−9.27***
	Psychological capital (PsyCap)		0.38	14.67***
Teaching competence	Occupational burnout	0.45	−0.12	−6.00***
	Psychological capital (PsyCap)		0.35	22.50***
	Self-efficacy		0.28	18.30***

*** Indicates *p* < 0.001, and the results are extremely statistically significant.

**Figure 2:**
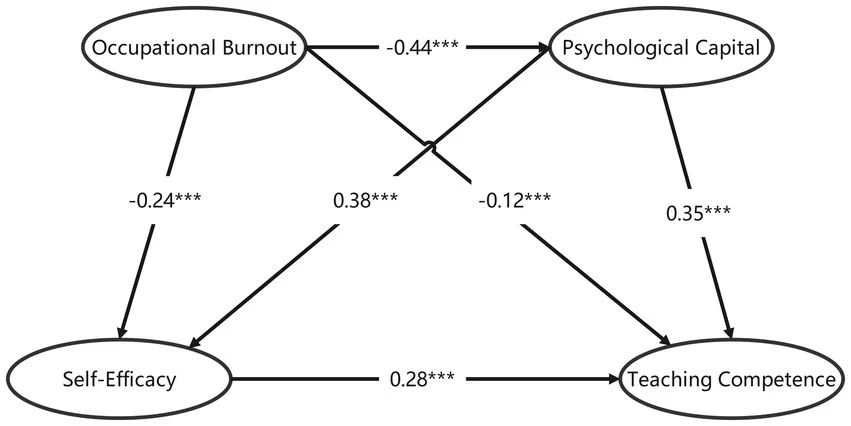
Mechanism model diagram of influence. *** Indicates *p* < 0.001, and the results are extremely statistically significant.

As can be seen from Table 2 and Figure 2, occupational burnout significantly negatively predicts psychological capital (PsyCap) (*β* = −0.44, *t* = −15.12, *p* < 0.001), indicating that the higher teachers’ occupational burnout level is, the more scarce their positive psychological resources such as hope and resilience are. Occupational burnout also exerts a significant negative predictive effect on self-efficacy (*β* = −0.24, *t* = −9.27, *p* < 0.001). Meanwhile, PsyCap significantly positively predicts self-efficacy (*β* = 0.38, *t* = 14.67, *p* < 0.001). The above results show that both PsyCap and occupational burnout play important roles in predicting teachers’ self-efficacy. Occupational burnout significantly negatively predicts teaching competence (*β* = −0.12, *t* = −6.00, *p* < 0.001), and PsyCap also has a significant positive predictive effect on teaching competence (*β* = 0.35, *t* = 22.50, *p* < 0.001), while self-efficacy significantly positively predicts teaching competence (*β* = 0.28, *t* = 18.30, *p* < 0.001). These results indicate that occupational burnout, PsyCap, and self-efficacy are all important predictive variables affecting the teaching competence of teachers in higher vocational colleges. Overall, the results of the regression analysis support the theoretical expectations regarding the direction of variable relationships in the research hypotheses, and lay a statistical foundation for the subsequent use of structural equation modeling (SEM) and the Bootstrap method to further test the paths among various variables.

### 4.4 Structural equation analysis

To further refine the goodness of fit of the chain mediation model of “occupational burnout → psychological capital → self-efficacy → teaching ability”, this paper uses structural equation modeling (SEM) to examine the influence relationships between variables, and uses AMOS 26.0 as the data analysis software.

#### 4.4.1 Model fit test

The results of the model fit index analysis are as follows: χ^2^/df = 2.87 (suitable range 1–3), GFI (Goodness of Fit Index) = 0.92, AGFI (Adjusted Goodness of Fit Index) = 0.90, NFI (Normed Fit Index) = 0.93, CFI (Comparative Fit Index) = 0.95, TLI (Tucker-Lewis Index) = 0.94, RMSEA (Root Mean Square Error of Approximation) = 0.042 (less than 0.05, indicating good model fit), SRMR (Standardized Root Mean Square Residual) = 0.038 (less than 0.05). All fit indices met or exceeded the critical value requirements, indicating that the established chain mediation structural equation model fits the data well and the model specification is valid.

#### 4.4.2 Multiple mediation effect test

As shown in Table 3, the results of the direct effect test indicate that occupational burnout has a significant negative direct impact on teaching competence (*β* = −0.12, *p* < 0.001), accounting for 19.42% of the total effect. This finding suggests that the higher the level of occupational burnout among teachers in higher vocational colleges, the lower their teaching competence, thus supporting Research Hypothesis H1.

**Table 3:** Results of multiple mediation effect test (Bootstrap = 5,000).

Effect type	Mediation path	Standardized regression coefficient (*β*)	Standard error (SE)	95% confidence interval (CI)	Proportion of total effect (%)	*p*-value
Direct effect	Occupational Burnout → Teaching Competence (H1)	−0.12	0.02	[−0.16, −0.08]	19.42	<0.001
Indirect effect 1	Occupational Burnout → Psychological Capital → Teaching Competence (H2)	−0.22	0.03	[−0.28, −0.16]	35.43	<0.001
Indirect effect 2	Occupational Burnout → Self-Efficacy → Teaching Competence (H3)	−0.18	0.02	[−0.22, −0.14]	29.03	<0.001
Indirect effect 3 (serial)	Occupational Burnout → Psychological Capital → Self-Efficacy → Teaching Competence (H4)	−0.10	0.02	[−0.14, −0.06]	16.12	<0.001
Total indirect effect	H2 + H3 + H4	−0.50	0.05	[−0.58, −0.42]	80.58	<0.001
Total effect	Direct Effect + Indirect Effect	−0.62	0.05	[−0.68, −0.56]	100	<0.001

For the single mediation effect tests, the mediating effect of psychological capital (PsyCap) is significant (*β* = −0.22, *p* < 0.001), contributing 35.43% to the total effect. Teachers’ occupational burnout impairs their positive psychological resources such as hope, resilience, and other related components (i.e., PsyCap). A lack of PsyCap reduces teachers’ confidence in addressing classroom challenges, thereby undermining their teaching performance. Therefore, Research Hypothesis H2 is supported.

The single mediating effect of self-efficacy is also significant (*β* = −0.18, *p* < 0.001), accounting for 29.03% of the total effect. This implies that occupational burnout erodes teachers’ teaching confidence, diminishes their willingness to engage in teaching innovation and investment, and consequently hinders their teaching competence. Thus, Research Hypothesis H3 is validated.

Regarding the chain mediation effect test, the serial mediating path of “occupational burnout → psychological capital → self-efficacy → teaching competence” is statistically significant (*β* = −0.10, *p* < 0.001), explaining 16.12% of the total effect. This demonstrates that occupational burnout impairs teachers’ PsyCap first, which in turn lowers their self-efficacy in teaching, and ultimately exerts an indirect restrictive effect on the development of their teaching competence. Hence, Research Hypothesis H4 is confirmed.

In summary, the total effect of occupational burnout on the teaching competence of teachers in higher vocational colleges is *β* = −0.62. Among this, the indirect effect (*β* = −0.50) accounts for 80.58%, while the direct effect (*β* = −0.12) accounts for 19.42%. These results indicate that the relationship between occupational burnout and teaching competence among teachers in higher vocational colleges is characterized by “a dominance of indirect effects supplemented by direct effects” and the coexistence of multiple influencing paths.

## 5 Discussion

The hypotheses on the relationships between occupational burnout, psychological capital (PsyCap), self-efficacy and teaching competence were empirically tested through correlation analysis, regression analysis and the Bootstrap multiple mediation effect tests using the data obtained in this study. The findings of the four research hypotheses are discussed in detail below in theoretical and practical terms.

### 5.1 The negative impact of occupational burnout on the teaching competence of teachers in higher vocational colleges

Hypothesis H1 was highly confirmed: occupational burnout adversely impacts on the teaching competence of teachers in higher vocational colleges significantly (*β* = 0.12, *p* < 0.001), and its direct influence explained 19.42 percent of the overall influence. This discovery can be used to provide empirical verification of how occupational burnout attacks teachers teaching abilities in three dimensions, namely, emotional exhaustion, depersonalization, and lessened personal achievement (Maslach and Jackson, 1981). As per descriptive statistics, the level of teachers occupational burnout was found to be 2.48 ± 0.59 (low to moderate) indicating that it had already affected their ability to teach. The underlying reason behind all this is that teachers in higher vocational colleges, not only are they loaded with dual duties of both theoretical instruction and practical training, but they are also burdened by many cumbersome tasks like applying to projects and concluding them and managing students. Excessive work over long periods is likely to lead to emotional exhaustion resulting in their diminished competence in teaching (Pan et al., 2023). It can be regarded as a phenomenon of teachers psychological capital drain and distortion of teaching behavior. Due to emotional exhaustion, teachers cannot make classrooms lively; due to depersonalization, teacher-student relations have become strained; due to diminished personal accomplishment, teachers do not become motivated to increase the quality of their teaching. The joint action of the three factors brings about a vicious cycle of insufficient investment-poor teaching effectiveness-impaired confidence in terms of teaching competence (Skaalvik and Skaalvik, 2020). It is noteworthy that this conclusion emphasizes the role of occupational burnout as one of the inhibitors of teachers competence, and more importantly, it offers direct empirical justification to prioritize teachers mental health and reduce occupational burnout in higher vocational colleges.

### 5.2 The mediating role of psychological capital

The research hypothesis H2 was highly confirmed and it is shown that psychological capital (PsyCap) has a strong mediating role between occupational burnout and competence in teaching (*β* = −0.22, *p* < 0.001), which explains 35.43 percent of the overall outcome. PsyCap being a positive psychological resource consisting of hope, optimism, resilience, and self-confidence, is one of the most vital psychological assets of teachers, enabling them to work under workplace pressure without losing their teaching efficacy (Luthans et al., 2010). Occupational burnout destroys the positive psychological qualities of a teacher and removes the psychological drive in teachers to overcome obstacles in routine teaching activities, which inevitably results in diminishing competencies in teaching (Ferradás et al., 2019). It is important to underline the fact that the relationship between PsyCap and teaching competence (*r* = 0.52, *p* < 0.01) is quite high in comparison with other variables, showing that PsyCap plays an important supportive function in enhancing teaching competence. Facing such situations as the burden of a big load of practical teaching responsibilities and unequal academic performance of students in higher vocational colleges, teachers must urgently have solid PsyCap as a psychological foundation. High PsyCap teachers have both the optimism and flexibility to meet a challenge in the classroom. These results will not only enhance the knowledge on how teachers occupational burnout influences the mechanisms but also confirm that PsyCap serves as a reservoir of positive psychological resources capable of alleviating the negative consequences of occupational burnout.

### 5.3 The mediating role of self-efficacy

Hypothesis H3 was strongly supported, confirming that self-efficacy plays a significant mediating role between job burnout and teaching ability (*β* = −0.18, *p* < 0.001), explaining 29.03% of the overall effect. This conclusion is supported by both Bandura’s self-efficacy theory (Bandura, 1997a). Providing a complete theoretical basis for the mediating path. Emotional exhaustion and job stress caused by Occupational Burnout will reduce teachers’ Self-Efficacy (perception of Teaching Competence); low Self-Efficacy will lead teachers to avoid teaching challenges and reduce teaching investment, ultimately lowering Teaching Competence, which directly supports the pathway of “Occupational Burnout → Self-Efficacy → Teaching Competence”. In the teaching and learning field of higher vocational colleges, the concept of self-efficacy plays a unique practical role. Vocational higher education attaches great importance to practice and application, and teachers have to deal with complex teaching situations. However, low self-efficacy caused by occupational burnout will have an adverse effect on teachers’ ability to manage the classroom and solve problems. The correlation analysis results show that the correlation coefficient between the two variables is r = 0.46, indicating that teaching confidence is the key factor connecting teachers’ psychological state and teaching efficacy. This observation shows that improving self-efficacy is a breakthrough intervention point that can interrupt the transmission chain that leads to occupational burnout affecting teaching efficacy and set specific goals for higher vocational education institutions to formulate and implement teacher vocational education ability training courses.

### 5.4 The chain mediating role of self-efficacy and psychological capital

Research Hypothesis H4 was strongly supported: the chain mediating effect of the path “occupational burnout → psychological capital → self-efficacy → teaching competence” was significant (*β* = −0.10, *p* < 0.001), accounting for 16.12% of the total effect. This constitutes one of the most important conclusions of this study, as it elaborates the in-depth transmission mechanism through which occupational burnout exerts a gradual impact on teaching competence. Specifically, occupational burnout first impairs teachers’ psychological capital; the resulting deficiency in psychological capital then leads to a decline in self-efficacy; ultimately, the chain reaction of “psychological capital depletion → self-efficacy decline” indirectly constrains teaching competence (Hu and Yeo, 2020). In this study, the chain mediating Role of the path “occupational burnout → psychological capital → self-efficacy → teaching competence” verifies the applicability of the Conservation of Resources Theory and Social Cognitive Theory among teachers in higher vocational colleges, and also confirms the theoretical logic of the internal hierarchical transmission of “psychological capital (resource reserve) → self-efficacy (belief formation) → teaching competence (behavioral performance)” (Zhang Q. et al., 2023). The verification of the chain mediating effect carries profound implications. Theoretically, it breaks through the limitations of single mediating effects and clarifies the interaction mode between psychological capital and self-efficacy: psychological capital serves as the “fundamental psychological strength” and constitutes the foundation for the development of self-efficacy, while self-efficacy represents the “cognitive belief about ability” that transforms psychological resources into actual teaching performance. From a practical perspective, the intervention strategies implemented by higher vocational colleges must simultaneously achieve two goals: “cultivating psychological capital” and “shaping self-efficacy.” On the one hand, colleges should provide mental health education to enrich teachers’ psychological qualities; on the other hand, they need to offer feedback on teaching performance to consolidate teachers’ confidence in teaching. Only by doing so can a positive mechanism of “psychological capital → self-efficacy” be established, thereby better interrupting the negative impact of occupational burnout on teaching competence through the mediating role of self-efficacy.

In summary, this study comprehensively reveals the pathways through which Occupational Burnout restricts the Teaching Competence of teachers in Chinese higher vocational colleges. The findings of this study not only expand the theoretical understanding of the correlation between teachers’ occupational burnout and teaching competence but also lay a reliable empirical foundation for higher vocational colleges to formulate refined and multi-dimensional teacher support policies.

## 6 Research limitations

Although this study reveals the core mechanism through which occupational burnout affects the teaching competence of teachers in higher vocational colleges, it still has the following limitations:

First, the study adopts a cross-sectional survey design, which can only verify the correlation between variables but cannot clarify the causal sequence among them, making it difficult to accurately judge the dynamic influence process of occupational burnout, psychological capital, and self-efficacy. Second, the study does not explore the role of moderating variables in depth. Factors that may affect the strength of the mediating paths, such as teachers’ years of teaching, professional titles, and college types, are not included in the analytical framework, failing to fully reveal the boundary conditions of the effects. These limitations point out the direction for future research. Subsequent studies can be improved by adopting longitudinal tracking design, expanding the sample scope, incorporating moderating variables, and using multi-source data collection methods.

## 7 Conclusion

This study takes teachers in higher vocational colleges as the research objects, systematically verifies four research hypotheses through empirical analysis, and reveals the influence mechanism of occupational burnout on teaching competence. The findings indicate that occupational burnout not only exerts a direct negative impact on teachers’ teaching competence, but also produces indirect effects through the single mediating paths of self-efficacy and psychological capital as well as the chain mediating path of “psychological capital → self-efficacy”. These results not only enrich the theoretical research on the relationship between teachers’ occupational burnout and teaching competence, providing new empirical evidence for understanding the complex correlation between them, but also offer practical guidance for the construction of teacher teams in higher vocational colleges. Higher vocational colleges can block the transmission path of burnout and improve teachers’ teaching competence by implementing multi-dimensional strategies such as alleviating teachers’ occupational stress, fostering positive psychological capital, and enhancing teaching self-efficacy.

## Data Availability

The raw data supporting the conclusions of this article will be made available by the authors, without undue reservation.
