# Correction: The impact of occupational burnout on teaching competence of teachers in Chinese higher vocational colleges: the mediating role of self-efficacy and psychological capital

**DOI:** 10.3389/fpsyg.2026.1905394

**Published:** 2026-08-12

**Authors:** Hailing Xie, Cheng Huang, Qing Zhang

**Affiliations:** 1 School of Education, Yangzhou University, Yangzhou, Jiangsu, China; 2 Department of Basic Courses, Jiangsu College of Tourism, Yangzhou, Jiangsu, China; 3 Hertfordshire College, Changzhou Institute of Technology, Changzhou, Jiangsu, China; 4 School of Law, Yangzhou University, Yangzhou, Jiangsu, China

**Keywords:** higher vocational colleges, occupational burnout, psychological capital, self-efficacy, teaching competence

Affiliation “Department of Basic Courses, Jiangsu College of Tourism, Yangzhou, Jiangsu, China” was omitted from the paper. This affiliation has now been added for author Hailing Xie.

The original version of this article has been updated.

